# Correlating Noble Rot Infection of Garganega Withered Grapes with Key Molecules and Odorants of Botrytized Passito Wine

**DOI:** 10.3390/foods8120642

**Published:** 2019-12-04

**Authors:** Barbara Simonato, Marilinda Lorenzini, Michela Cipriani, Fabio Finato, Giacomo Zapparoli

**Affiliations:** 1 Dipartimento di Biotecnologie, Università degli Studi di Verona, Strada Le Grazie 15, 37134 Verona, Italy; barbara.simonato@univr.it (B.S.); lorenzini.marilinda@gmail.com (M.L.); 2 Unione Italiana Vini, Viale del lavoro 8, 37135 Verona, Italy; m.cipriani@uiv.it (M.C.); f.finato@uiv.it (F.F.)

**Keywords:** noble rot, withered grape, passito wine, aroma

## Abstract

Experimental passito wines with different percentages of naturally noble-rotten grapes of the Garganega variety were analyzed to evaluate key molecules and odorants related to the typical aroma and sensory profile of botrytized passito wine. Remarkable changes in the concentration of 1-octen-3-ol, 4-terpineol, benzaldehyde, *N*-(3-methylbutyl)acetamide, and sherry lactone 1 and 2 were observed between sound and noble-rotten wines. Wines were perceived to be different for floral, honey, figs, apricot, and caramel scents. By partial least square regression these descriptors were well correlated to samples. An important positive contribution of sherry lactones, *N*-(3-methylbutyl)acetamide, vanillin, benzaldehyde, and γ-butyrolactone to honey, apricot, and caramel was observed. It is conceivable that oxidative effects of *Botrytis cinerea* infection play an important role in the genesis of these chemical and sensory aroma markers. This study provides a predictive tool for winemakers that use natural grape withering to produce wines whose aroma profile is not standardized due to the seasonal variation of noble rot incidence.

## 1. Introduction

The positive impact of endophytic grape infection of *Botrytis cinerea*, called noble rot, on properties of sweet wines, like the famous Sauternes, Tokaji Aszu, Trockenbeerenauslese, and Beerenauslese, is well known [1,2]. The effects of noble rot on wine quality were also investigated in Italian passito wines such as Amarone, Recioto, and Vin Santo, produced from off-vine withered grapes [3,4,5,6]. Recently, the chemical and sensory properties of Chardonnay botrytized wine from artificially infected grapes has been analyzed [7].

Italian passito wines are not deliberately produced from botrytized grapes and the impact of noble rot highly depends on the amount of infected grapes used in vinification. Since the frequency of this infection varies greatly according to the seasonal conditions, the contribution of noble rot on the aroma profile of passito wine is very variable [8]. For this reason, the use of controlled botrytization in post-harvest conditions under a semi-controlled environment to enhance the positive effects of noble rot on wine quality has been investigated [8]. The impact of artificially induced grape botrytization on the chemical and sensory properties of Recioto di Soave wine has been documented [5,6]. However, most passito wine producers still use natural withering of grapes and prefer to obtain the wine according to the traditional protocol ensuring the authenticity of their wines although the aroma profile is not standardized due to the seasonal variation of noble rot incidence.

Potential key odorant molecules related to the aroma of botrytized wine have been identified in different types of wine such as sweet botrytized wines from the Bordeaux region [9], Tokaji Aszu’ wine [10], sweet Fiano wine [11], and sweet Chardonnay wine [7]. In the case of Italian passito wine obtained from noble-rotten grapes, besides mushroom smelling compound 1-octen-3-ol, other molecules with floral attributes such as monoterpens (i.e., 4-terpineol) and with vinegar notes such as acetamides (i.e., *N*-(3-methylbutyl)acetamide) are recognized to be indicative of mold infection in withered grapes [3,4,5,6]. Nevertheless, the correlation of noble rot infection with these aroma markers on passito wine is still unclear, and the relationship between the fungus and passito wine quality needs further investigation.

With this aim, natural noble-rotten grapes of the Garganega variety (white grape) were selected and used together with sound ones in experimental vinifications. Partial least square (PLS) regression was applied to establish the relationship between samples, sensory attributes, and aroma molecules. This study attempts to predict typical odorants of botrytized passito wine from aroma molecules according to the different incidence of noble rot infection on withered grapes.

## 2. Materials and Methods

### 2.1. Grape Sampling and Vinifications

**Figure 1:**
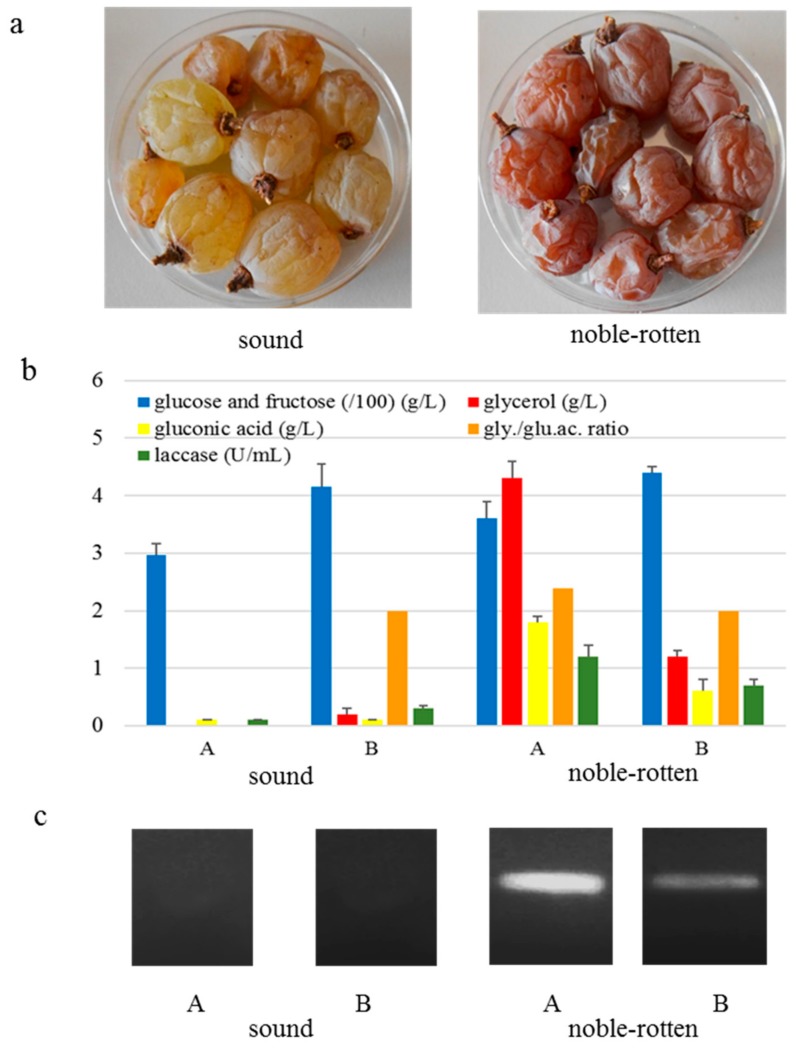
Sound and noble-rotten berries visually selected for the two vinifications A and B (**a**), glucose and fructose content, glycerol, gluconic acid, their ratio and laccase activity (bars are standard deviation) (**b**), and amplification of 196 bp *Botrytis cinerea* specific band (**c**) determined on the resulting musts.

It was carried out on two vinification groups (A and B) using Garganega grapes of two different localities of the Soave-Gambellara winemaking area (Italy). This variety is used to produce passito wines such as Recioto di Soave Denominazione di origine controllata e garantita (DOCG), Recioto di Gambellara DOCG, and Vin Santo di Gambellara Denominazione di origine controlla (DOC). DOCG and DOC are designations of quality according to the rules of the production of Italian wines. Grapes were withered in natural conditions. After the harvest, grape clusters were manually laid down on plastic boxes placed in a fruit-drying room (fruttaio) without controlled temperature and relative humidity conditions according to the traditional surmaturation technique. The withering lasted about four months to allow the dehydration up to a weight loss of 40–45%. At the end of withering process, sound and noble-rotten grapes were recognized by visual inspection. Sound berries were swollen or partially shriveled with homogenous yellowish to amber skin, while those affected by noble rot were shriveled with light to dark brown skin color (Figure 1).

The grape crushing was carried out using an electric small crusher-stemmer in a winery room without controlled temperature (room temperature ranged from 8 to 12 °C). Sulfur dioxide (50 mg L^−1^) was added on the resulting juices collected in clean vessels to inhibit indigenous microrganisms and prevent oxidation. These grape crushing conditions were similar for both vinifications. Vinifications A were produced from single sound and noble-rotten berries, separated manually into two batches and crushed singly to obtain homogenous musts. “Sound” wine (A-S) was produced from only sound berries, while “noble-rotten” wines (A-N20 and A-N40) were produced mixing 20 and 40% botrytized and 80 and 60% sound must, respectively. Vinifications B were produced from sound and noble-rotten bunches manually selected according to the predominant presence of sound or noble-rotten berries in each bunch, respectively. Sound and noble-rotten bunches were separately crushed to obtain the respective musts. “Sound” wine (B-S) was produced by must only obtained from sound bunches, while “noble-rotten” wines (B-N50) was produced mixing 50% sound and 50% botrytized must. The vinifications B using grape bunches instead of single berries was carried out to simulate at the crushing a possible winery condition in term of sanitary status of withered grapes.

Each trial of the two vinification groups (A and B) was carried out in triplicate doing separate vinifications in a final volume of 12 L (vinifications A) and 40 L (vinifications B). The commercial yeast strain *Saccharomyces cerevisiae* EC1118 (Lallemand Inc, Montréal, Canada) was added at the concentration of approximately 10^6^ cfu mL^−1^ in all trials to drive the alcoholic fermentation according to manufacturer’s instructions. The fermentation was conducted in glass carboy (20 and 54 L for vinifications A and B, respectively) with airlock caps placed in a winery room without controlled temperature (room temperature ranged from 7 to 13 °C). After the fermentation, wines were de-vatted and decanted at 4 °C for three days, then stabilized adding 50 mg L^−1^ SO_2_ before the analysis.

### 2.2. Analysis of Grape Must

Glucose, fructose, gluconic acid, and glycerol in must were determined using commercial enzymatic kits (Megazyme, Bray, Ireland). Laccase activity was determined using as substrate five mM 2,6-dimethoxyphenol (Sigma-Aldrich, St. Louis, MO, USA) according to Tosi et al. [4] and expressed as unit per mL (U mL^−1^), where one unit corresponds to the amount of enzyme converting one μmol of substrate over one min.

The detection of *B. cinerea* in the grapes was confirmed by performing a species-specific polymerase chain reaction (PCR) analysis using as template DNA extracted from an amount of 50 g of berries. DNA extraction was carried out according to Rezaian and Krake [12], while PCR assay was performed using Bot-F and Bot-R primers as previously described by Lorenzini and Zapparoli [13].

### 2.3. Analysis of Wines

Wines were characterized by parameters such as reducing sugars, total acidity, ethanol and total dry extract according to methods proposed by O.I.V. International Oenological Codex 2010.

The volatile compounds were determined by gas chromatography—mass spectrometry (GC-MS). The extraction of these compounds was carried out by Solid Phase Extraction (SPE) using the methods described by Zapparoli et al. [14]. Briefly, the extraction was carried out one time for each sample on an automated solid phase extraction apparatus with an Aspec XL Sample Processor (Gilson Inc., Middleton, WI, USA). The cartridges were sequentially conditioned with methanol (9.5 mL) and distilled water (19 mL). 1-heptanol, as internal standard solution (500 µg L^−1^), was added to 38 mL of wine samples diluted 1:2 with distilled water, then loaded onto the cartridge. The residue was washed with distilled water (19 mL), and compounds were eluted with dichloromethane (9 mL). The solution was dried with Na_2_SO_4_ and concentrated to 0.4 mL by nitrogen flow stream. The extraction was carried out one time for each sample. The extract was injected in splitless mode (volume 2 μL) onto a gas chromatography–mass spectrometry using 6890 Network GC systems coupled with a 5975 XL EI/CI MSD (Agilent Technologies, Santa Clara, CA, USA). Separation was carried out with a DB-Wax Bonded PEG fused silica capillary column (60m × 320 μm i.d. × 0.25 μm filn thickness; Agilent Technologies). The temperature program was follows: 50 °C for 4 min, raising to 240 °C at a rate of 4 °C min, then 16 min at 240 °C. The injector temperature was 250 °C and ion source temperature was 230 °C.

National Institute Standards and Technology (NIST) data bank and co-injection of pure references standards (Sigma-Aldrich) were used for the identification of compounds. Quantification of molecules was carried out using an internal standard method and the response factors were calculated by calibration curves in a hydroalcoholic solution, containing 12% *v*/*v* ethanol with 5 g L^−1^ tartaric acid, like a model wine. For quantification of compounds for which commercial standards were not available, the response factors of those with similar chemical structures were utilized.

The odor activity values (OAVs) were calculated as the ratio between the measured concentration of a substance in the wine and its odor threshold, when available.

### 2.4. Sensory Analysis

Descriptive analysis of experimental wines was carried out in sessions by eight experts of passito wines. All judges had wine tasting experiences. In training sessions aroma reference standards were discussed and, by consensus, the most appropriate terms were selected to define aroma differences among the wines. In this phase of the analysis, the panelists identified 18 descriptors. The descriptors were then reduced to 10 (aroma intensity, floral, almond, honey, figs, apricot, vegetal, resinous, vanilla, and caramel) after the elimination of non-pertinent attributes by using statistical methods described by International Organization for Standardization (ISO) 11035 [15]. These descriptors were recognized as typical odorants of passito wine from Garganega grapes (Recioto di Soave and Gambellara, and Vin Santo di Gambellara). It was decided to assign to each descriptor a score using a 10-point scale (from unnoticeable to very strong). In a second type of session, the five wines were evaluated in replicates using a randomized complete block design. The panelists were provided with about 30 mL of wine in coded standard clear wine glasses (viognier glasses), covered with a plastic dish. They were instructed to rinse three times with water between samples. In 1 h sessions, panelists smelled and tasted each different wine, with replication. All breaks between individual samples and replicated sample sets were taken to avoid fatigue. Presentation order was randomized within each scoring repetition. Assessment took place in a standard sensory-analysis chamber, equipped with separate booths, at room temperature (20–22 °C).

### 2.5. Statistical Treatment of Data

Variance analysis (ANOVA) was used for the wine compounds and sensory scores to evaluate the differences between and within the samples. Tukey’s multiple comparison test (Tukey HSD) was applied to determine significant differences between samples and chemical compounds or sensory scores. The partial least-squares regression model was carried out using XLSTAT (Addinsoft SARL, Paris, France) to explore the correlations between wine, aroma compounds (X-variables), and olfactive descriptors (Y-variables). The model was cross validated using the Jackknife testing method (LOO). Model quality was evaluated by Q2, R2Y, and R2X cumulative scores. Components t and u are related to X and Y scores, respectively.

## 3. Results

### 3.1. Grape Analysis

Data on chemical and enzymatic key parameters of noble rot infection on grapes revealed differences among the two grape samples for the vinifications A and B (Figure 1a,b). Grapes A had a greater difference of glucose and fructose concentration, glycerol and gluconic acid, and laccase activity between sound and infected berries than grapes B. The molecular detection of *B. cinerea* by PCR confirmed its absence in sound grapes, while the amplification of the band indicated the presence of low fungal infection on noble-rotten grapes (Figure 1c).

### 3.2. Volatile Composition of Wines

Concentrations of total dry extract, residual reducing sugars, total acidity and ethanol (Table S1) were considered standard values for Recioto di Soave DOCG or Recioto di Gambellara DOCG, according to their respective regulations of production.

Remarkable changes in the concentration of some molecules such as 1-octen-3-ol, 4-terpineol, benzaldehyde, *N*-(3-methylbutyl)acetamide, sherry lactone 1 and 2 were observed between sound and noble-rotten wines of both vinifications (Table 1).

All these compounds increased over 100% in wines produced from noble-rotten grapes compared with sound wines. The significant increase of the content on ethyl 4-hydroxybutyrate, citronellol, vanillin, and γ-butyrolactone, and decrease of ethyl lactate, 1-hexanol, 3-methylthiopropanol, octanoic acid, and decanoic acid in noble-rotten wines was also observed.

Other molecules varied between sound and noble-rotten wines, but differently in the two vinifications. In vinifications A, acetate esters (hexyl acetate, isoamyl acetate, β-phenylethyl acetate, and ethyl 2-phenylacetate) varied according to the percentage of noble-rotten grapes, while only β-phenylethyl acetate was significantly higher in the B-S wine than the B-N50 wine. Significant changes in the content of trans-3-hexenol, β-damascenone, 4-carboethoxy-γ-butyrolactone and furfural were found between the B-S and B-N50 wines. The trend of benzyl alcohol was opposite in the two vinifications because it decreased in A-N20 and A-N40, but increased in B-N50.

The changes in volatile compositions of wines linked to noble rot compared to sound wines determined variations on OAV values of important molecules. It was observed that 1-octen-3-ol (mushroom note) was near its threshold (25 µg L^−1^) [16] only in A-N40 (OAV = 0.9). The OAV of isoamyl acetate (banana note, threshold of 30 µg L^−1^) [17]) strongly decreased from 29 in A-S wine to 14 and 10 in A-N20 and A-N40, respectively. A decrease of OAV of octanoic acid (fatty, cheese scent, threshold of 500 µg L^−1^) [17] was observed in all noble-rotten wines (from 6.6 in A-S wine to 4.8 in A-N20 and A-N40, and from 3.6 in B-S to 2.9 in B-N50), while OAV of hexanoic acid (fatty, cheese scent, threshold of 420 µg L^−1^) [17] decreased only in A-N20 and A-N40 (from 6.1 in A-S to approximately 4.5 in A-20 and A-40). The OAV of β-damascenone (floral, sweet note, threshold of 0.05 µg L^−1^) [17] decreased in B-N50 (from 198 in B-S to 132 in B-N50). The OAV of 3-methylthiopropanol (potato and cooked vegetable note), that was 1.3 in B-S wine, decreased under its threshold (1000 µg L^−1^) [17] in B-N50.

### 3.3. Sensory Analysis and PLS-R Analysis

All descriptors, except almond, had an average score significantly different among wines (Table 2). 

In the wines of vinifications A, one or both noble-rotten wines were discriminated for aroma intensity, floral, honey, figs, apricot, vegetal, and caramel. The B-N50 was significantly different from B-S wine for aroma intensity, floral, honey, figs, apricot, resinous, and caramel. All noble-rotten wines had a lower score of floral scent and higher note of honey and caramel than the two sound wines, while only for figs and apricot A-N20 and A-S were not perceived as different.

**Figure 2:**
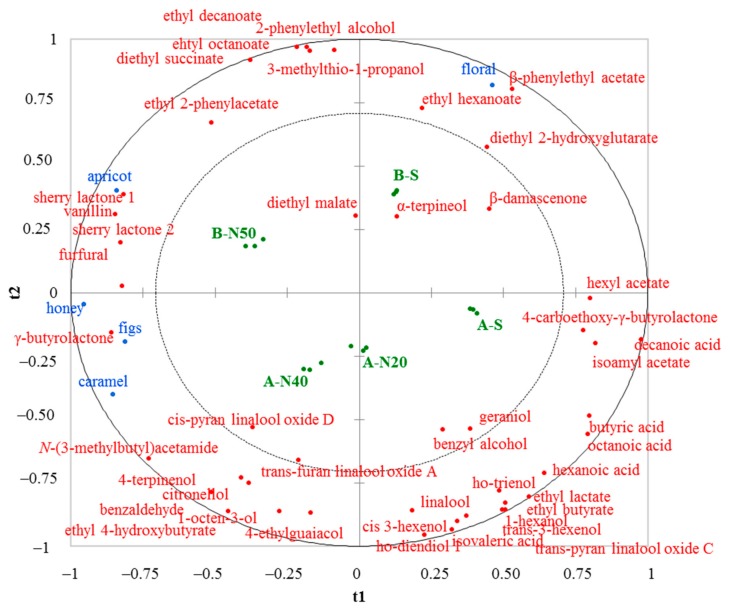
Bi-plot t1 and t2 components with wines (green spot), sensory descriptors (blue spots), and molecules (red spots).

It was carried out the PLS-R analysis to investigate the relationship between the descriptors and aroma molecules detected in the wines. Analysis performed by floral, honey, figs, apricot, and caramel furnished the best model quality with parameters cumulative Q2 of 0.422, 0.721, and 0.690, R2Y of 0.644, 0.851, and 0.879, and R2X of 0.287, 0.764, and 0.876 for the first three components, respectively. The correlation between wines, molecules and descriptors on bi-plot t1 and t2 is shown in Figure 2. 

The discrimination of wines according to noble rot grape infection was carried out mainly along t1 component (sound wines and noble-rotten wines were on the right and left, respectively), while wines produced by the two vinifications were separated along t2 component (wines A and B were below and up, respectively). Regarding the relationship between variable Y (descriptors), variable X (molecules), and wines, B-N50 wines were mainly related with apricot, sherry lactones and ethyl phenylacetate, A-N40 and A-N20 wines with caramel and some compounds including *N*-(3-methylbutyl)acetamide, 4-terpinenol, and benzaldehyde, and B-S with floral and phenylethyl acetate, ethyl hexanoate, and ethyl 2-hydroxyglutarate.

**Figure 3:**
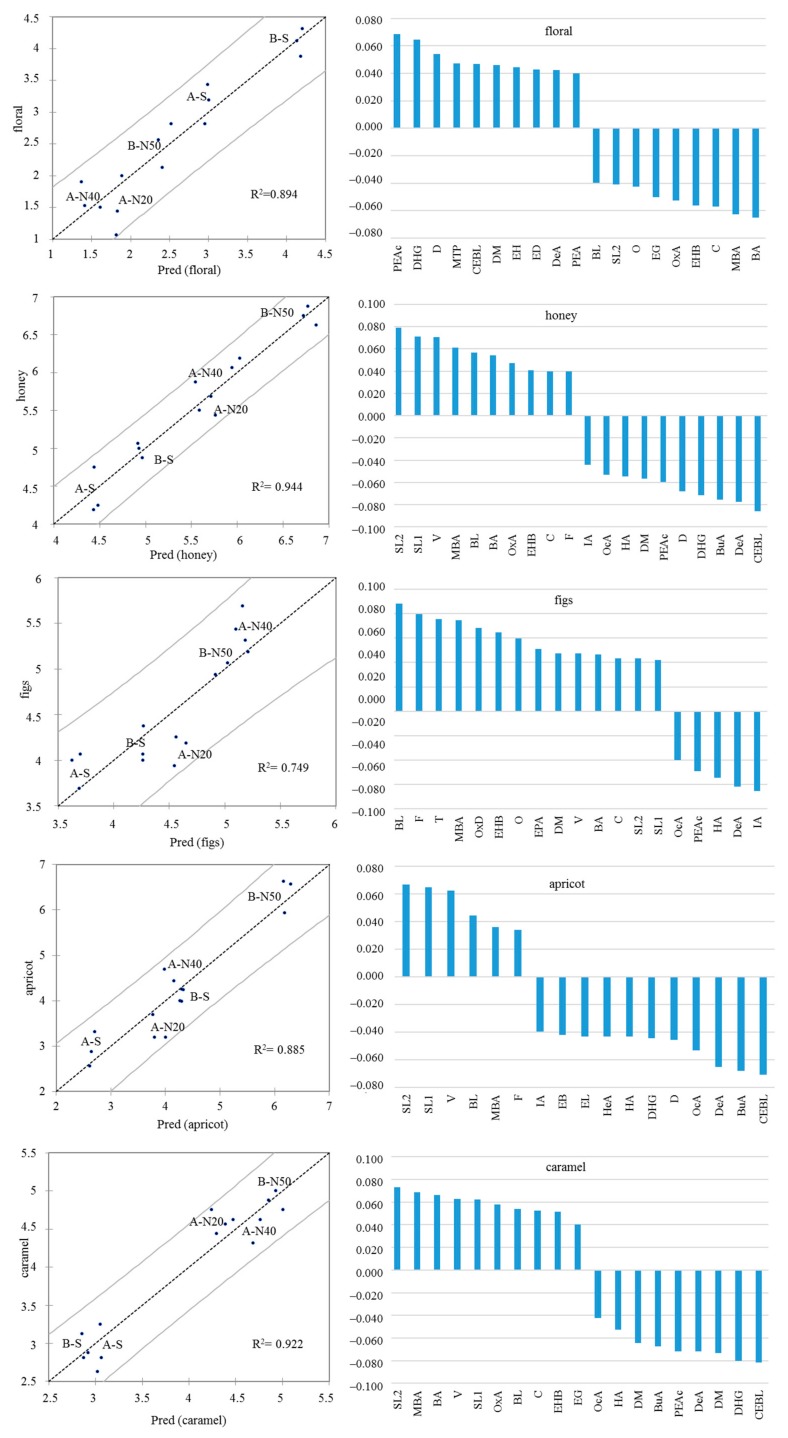
Partial least squares (PLS) regression (left) and standardized coefficients (right) by main volatile compounds for floral, honey, figs, apricot, and caramel. BA, benzaldehyde; BL, γ-butyrolactone; BuA, butyric acid; C, citronellol; CEBL, 4-carboethoxy-γ-butyrolactone; D, β-damascenone; DeA, decanoic acid; DHG, diethyl 2-hydroxyglutarate; DM, diethyl malate; EB, ethyl butyrate; ED, ethyl decanoate; EG, 4-ethylguaiacol; EL, ethyl lactate; EH, ethyl hexanoate; EHB, ethyl 4-hydroxybutyrate; EPA, ethyl 2-phenylacetate; F, furfural; IA, isoamyl acetate; HA, hexyl acetate; HeA, hexanoic acid; MBA, N-(3-methylbutyl)acetamide; MTP, 3-methylthio-1-propanol; O, 1-octen-3-ol; OcA, octanoic acid; OxA, trans-furan linalool oxide A; OxD, cis-Pyranic linalool oxide D; PEA, 2-phenylethyl alcohol; PEAc, β-phenylethyl acetate; SL1, sherry lactone 1; SL2, sherry lactone 2; T, 4-terpinenol; V vanillin.

The best prediction model results for sensory descriptors are presented in Figure 3.

The R2 value was the highest for honey (0.944) and lowest for figs (0.749). Correlation coefficients of the most positively and negatively correlated molecules to the five descriptors indicated the important positive or negative contribution of sherry lactones, *N*-(3-methylbutyl)acetamide, vanillin, benzaldehyde, γ-butyrolactone, and furfural, while less relevant was the contribution of 1-octen-3-ol and 4-terpineol (Figure 3).

## 4. Discussion

This study analyzed the changes in the volatile composition and sensory properties of passito wines related to the natural occurrence of the *B. cinerea* endophytic infection in grapes according to traditional withering protocol. 

Chemical and sensory analysis revealed that noble rot infection provided remarkable changes in the aroma and flavor of passito wine according to previous investigations [3,4,5,6,18]. The increase (over 100%) of some molecules such as sherry lactones, *N*-(3-methylbutyl)acetamide, 1-octen-3-ol, 4-terpinen-1-ol, and benzaldehyde was clearly linked to the fungal infection, so they can be considered markers of noble rot in passito wines. Similar behavior of these molecules has been observed in most passito wines such as Recioto di Soave and Amarone wine, previously analyzed by comparing healthy and botrytized wines [3,4,5,6,18]. Moreover, a great increase of 1-octen-3-ol, 4-terpinen-1-ol, benzaldehyde, and *N*-(3-methylbutyl)acetamide was also observed in sweet Fiano wine produced with botrytized withered grapes compared with the same wine obtained from fresh grapes [11]. If 1-octen-3-ol and benzaldehyde are known molecules associated with *Botrytis* metabolism [16,19], there is no evidence of the active metabolic role of *Botrytis* in the increase of 4-terpinen-1-ol and *N*-(3-methylbutyl)acetamide in grape must. It is conceivable that the increase of these compounds in botrytized grapes may be attributed to the effects of *Botrytis* infection on the physical and chemical properties of the berry. Since a high content of monoterpenes and *N*-(3-methylbutyl)acetamide has been associated with skin contact during must preparation [20,21], changes in berry skin properties due to mold infection could favor the increase of these compounds in botrytized wine. Further investigation is necessary to support this assumption.

The higher concentration of the two isomers of 4,5-dihydroxyhexanoic acid gamma-lactone (sherry lactone or solerol) in noble-rotten wines with respect to healthy wines could be related to oxidative processes promoted by the fungus. These lactones have been found in flor sherry [22,23], figs, and dates [24] and are generated by the solerone (5-oxo-4-hexanolide) pathway starting from the enzymatic oxidative deamination of glutamic acid 5-ethyl esters [25]. Considering that the key step of this pathway is fungal pyruvate carboxylases, including those of fermenting yeasts, but also the involvement of reducing enzymes, further investigation is necessary to understand the enzymatic role of *Botrytis* in favoring the synthesis of these lactones.

Regarding the odorants correlated with noble-rotten grapes, sensory analysis showed that *Botrytis* infection enhanced sensory notes, such as figs, honey, apricot, and caramel, which are common to other passito white wines (e.g., Vin Santo Toscano and Passito di Pantelleria) and sweet white wines produced in different countries [7,26,27]. Based on PLS analysis, these descriptors have been correlated with those molecules associated with noble rot. Wang et al. [7] carried out the PLS analysis of Chardonnay wines and found that thiols, C13-norisoprenoids, and lactones made a significant contribution to the dry apricot aroma of those obtained by noble-rotten grapes. The correlation of the honey note to *Botrytis* infection is in accordance with Ferreira et al. [28], who reported that a honey-like note was a key odorant related to the typical aroma of oxidation-spoiled white wine. The enhancing of oxidative reactions in grapes and wine due to fungal grape infection is well known [29]. Moreover, effects of fungal oxidation are congruent with the increased caramel scent in noble-rotten wine, a characteristic odorant of sweet fortified wines [30]. According to Etiévant [31], sherry lactones are odorless, while *N*-(3-methylbutyl)acetamide contribute a vinegar note. Seemingly, it is difficult to understand why these two molecules had high correlation coefficients for apricot, honey, and caramel. It cannot be excluded that possible masking, additive or synergic effects, previously described for some sensory wine descriptors [32], may contribute to the perception of the typical odorants of noble-rotten passito wine. Naturally, further investigation is needed to verify this assumption.

In conclusion, this study evidenced the correlation of the noble rot infection of Garganega withered grapes with molecules such as sherry lactones, *N*-(3-methylbutyl)acetamide, vanillin, benzaldehyde, and γ-butyrolactone, and odorants such as honey, apricot, and caramel. The accumulation of 1-octen-3-ol and 4-terpineol, known compounds associated with a fungal infection, seems to have a minor contribution to the organoleptic properties of the Recioto di Soave and Gambellara wines. These results suggest a possible specific effect of noble rot infection on aroma and sensory profile of this type of passito wine. Finally, through the experimental protocol described in the present study, the prediction of key sensory properties from the main aroma molecules detected in passito wine is proposed. This predictive tool is useful for winemakers that use the natural grape withering process. Obviously, this model needs to be improved by deeper analysis to evaluate the combined effects of *Botrytis* endophytic infection and the withering process, which are typical of passito wine.

## Figures and Tables

**Table 1:** Volatile compounds (µg L^−1^) of five passito wines obtained from sound (A-S and B-S) and noble-rotten (A-N20, A-N40 and B-50) grapes.

	A-S	A-N20	A-N40	B-S	B-N50
AAlcohols					
1-Hexanol	1978.3 ± 92.5 ^a^	1698.0 ± 76.7 ^b^	1633.0 ± 48.8 ^b^	435.7 ± 12.4 ^c^	370.7 ± 14.5 ^c^
*trans*-3-Hexenol	134.0 ± 10.1 ^a^	130.0 ± 4.6 ^a^	117.0 ± 10.4 ^a^	30.0 ± 1.7 ^b^	12.0 ± 4.4 ^b^
*cis*-3-Hexenol	17.3 ± 3.5 ^ab^	19.7 ± 3.5 ^a^	25.3 ± 8.5 ^a^	5.7 ± 1.2 ^bc^	3.0 ± 1.0 ^c^
Benzyl alcohol	728.7 ± 35.2 ^a^	396.3 ± 18.0 ^c^	494.3 ± 21.9 ^b^	187.0 ± 14.7 ^d^	463.7 ± 30.6 ^bc^
2-Phenylethyl alcohol	24703.0 ± 132.4 ^c^	24579.0 ± 66.1 ^c^	23765.3 ± 738.6 ^c^	42912.7 ± 146.2 ^a^	36545.0 ± 193.1 ^b^
3-Methylthio-1-propanol	153.0 ± 9.2 ^c^	112.7 ± 9.3 ^c^	106.0 ± 6.0 ^c^	1368.7 ± 57.8 ^a^	752.7 ± 35.9 ^b^
1-Octen-3-ol	4.7 ± 0.6 ^c^	14.7 ± 1.5 ^b^	23.7 ± 2.3 ^a^	0.7 ± 0.6 ^d^	3.3 ± 0.6 ^cd^
Esters					
Isoamyl acetate	876.7 ± 35.2 ^a^	435.7 ± 34.6 ^b^	300.3 ± 11.5 ^c^	335.0 ± 12.8 ^c^	306.3 ± 18.0 ^c^
Hexyl acetate	19.7 ± 3.1 ^a^	1.7 ± 0.6 ^b^	1.3 ± 0.6 ^b^	4.0 ± 1.7 ^b^	2.0 ± 1.0 ^b^
*β*-Phenylethyl acetate	73.7 ± 11.0 ^a^	15.7 ± 2.1 ^c^	4.3 ± 1.2 ^c^	86.3 ± 4.6 ^a^	47.7 ± 3.1 ^b^
Ethyl 2-phenylacetate	1.3 ± 0.6 ^c^	2.3 ± 0.6 ^bc^	4.3 ± 1.2 ^ab^	7.0 ± 1.0 ^a^	6.0 ± 1.7 ^a^
Ethyl butyrate	206.7 ± 8.4 ^a^	202.3 ± 5.9 ^a^	198.0 ± 10.1 ^a^	143.7 ± 10.6 ^b^	126.7 ± 7.1 ^b^
Ethyl hexanoate	436.3 ± 18.2 ^a^	341.3 ± 24.1 ^b^	341.0 ± 19.3 ^b^	423.7 ± 12.0 ^a^	437.7 ± 10.8 ^a^
Ethyl octanoate	130.0 ± 2.6 ^c^	89.0 ± 11.5 ^d^	101.3 ± 6.8 ^cd^	643.0 ± 23.9 ^a^	501.0 ± 12.0 ^b^
Ethyl decanoate	39.0 ± 6.1 ^c^	32.3 ± 6.7 ^c^	40.3 ± 7.2 ^c^	181.7 ± 13.1 ^a^	124.3 ± 7.5 ^b^
Ethyl lactate	2879.7 ± 40.5 ^a^	2571.7 ± 16.4 ^b^	2373.0 ± 33.0 ^c^	1096.7 ± 26.8 ^d^	628.0 ± 6.6 ^e^
Ethyl 4-hydroxybutyrate	6000.3 ± 198.0 ^d^	7388.7 ± 115.5 ^b^	9834.3 ± 99.2 ^a^	3379.3 ± 36.1 ^e^	6746.0 ± 59.6 ^c^
Diethyl 2-hydroxyglutarate	105.7 ± 8.4 ^b^	98.3 ± 9.9 ^b^	102.0 ± 11.1 ^b^	211.3 ± 7.8 ^a^	66.7 ± 8.6 ^c^
Diethyl malate	1312.0 ± 35.8 ^c^	1387.3 ± 28.2 ^c^	1792.3 ± 33.9 ^b^	1972.7 ± 29.1 ^a^	1335.0 ± 34.7 ^c^
Diethyl succinate	967.7 ± 30.1 ^d^	1111.3 ± 9.1 ^c^	1200.3 ± 16.5 ^c^	3151.0 ± 23.6 ^a^	2913.0 ± 92.6 ^b^
Acids					
Isovaleric acid	692.7 ± 16.3 ^a^	698.7 ± 17.0 ^a^	730.0 ± 23.3 ^a^	417.0 ± 5.6 ^b^	415.0 ± 53.9 ^b^
Butyric acid	682.0 ± 26.5 ^a^	593.3 ± 10.7 ^b^	619.7 ± 14.3 ^b^	575.3 ± 30.3 ^b^	464.0 ± 6.2 ^c^
Hexanoic acid	2593.0 ± 23.3 ^a^	1959.0 ± 55.7 ^b^	1947.7 ± 23.5 ^b^	1204.0 ± 60.9 ^c^	1172.7 ± 30.6 ^c^
Octanoic acid	3326.7 ± 37.1 ^a^	2412.7 ± 25.1 ^b^	2337.0 ± 16.6 ^b^	1828.7 ± 25.9 ^c^	1471.3 ± 35.9 ^d^
Decanoic acid	754.7 ± 11.5 ^a^	533.7 ± 10.1 ^b^	467.0 ± 12.5 ^c^	548.7 ± 4.5 ^b^	300.3 ± 3.1 ^d^
Terpens					
*trans*-Furanic linalool oxide A	1.3 ± 0.6	1.7 ± 0.6	1.7 ± 0.6	0.3 ± 0.6	1.3 ± 0.6
*trans*-Pyranic linalool oxide C	34.0 ± 4.6 ^a^	32.7 ± 6.7 ^a^	32.3 ± 3.5 ^a^	7.7 ± 1.2 ^b^	11.3 ± 0.6 ^b^
*cis*-Pyranic linalool oxide D	0.7 ± 0.6	1.3 ± 0.6	2.3 ± 0.6	0.7 ± 0.6	1.0 ± 1.0
Linalool	10.3 ± 0.6 ^a^	10.0 ± 1.0 ^a^	10.3 ± 2.5 ^a^	2.7 ± 1.5 ^b^	2.3 ± 0.6 ^b^
α-Terpineol	2.7 ± 0.6	3.0 ± 1.0	2.7 ± 1.2	3.7 ± 1.2	2.7 ± 0.6
4-Terpinenol	8.3 ± 1.5 ^d^	73.7 ± 3.2 ^b^	156.0 ± 6.1 ^a^	15.0 ±2.0 ^d^	25.0 ± 3.6 ^c^
Citronellol	6.3 ± 1.2 ^ab^	9.0 ± 1.0 ^a^	9.3 ± 0.6 ^a^	5.3 ± 1.2 ^b^	7.0 ± 1.7 ^ab^
Geraniol	2.7 ± 0.6	2.0 ± 1.0	2.3 ± 0.6	1.3 ± 0.6	1.3 ± 0.6
ho-Diendiol 1	85.3 ± 7.5 ^b^	89.3 ± 8.7 ^b^	106.7 ± 6.1 ^a^	16.3 ± 5.0 ^c^	21.7 ± 3.2 ^c^
ho-trienol	19.0 ± 2.0 ^a^	16.7 ± 3.8 ^a^	15.7 ± 7.1 ^a^	3.7 ± 1.2 ^b^	2.3 ± 1.2 ^b^
Carbonyl compounds					
*β*-Damascenone	7.7 ± 1.2 ^ab^	7.0 ± 1.0 ^b^	8.0 ± 1.0 ^ab^	10.0 ± 1.0 ^a^	6.0 ± 0.0 ^b^
Benzaldehyde	32.3 ± 3.8 ^c^	167.3 ± 8.5 ^a^	180.7 ± 10.5 ^a^	23.7 ± 3.1 ^c^	83.3 ± 2.1 ^b^
Furfural	42.7 ± 4.7 ^d^	77.0 ± 5.2 ^c^	105.0 ± 4.6 ^a^	86.0 ± 4.4 ^bc^	92.3 ± 6.5 ^ab^
Lactones					
*γ*-Butyrolactone	1253.7 ± 56.4 ^b^	1299.7 ± 21.5 ^b^	1580.3 ± 91.8 ^a^	1253.7 ± 12.9 ^b^	1626.7 ± 16.2 ^a^
4-Carboethoxy-*γ*-butyrolactone	353.7 ± 6.4 ^ab^	330.0 ± 24.3 ^b^	323.7 ± 21.8 ^b^	374.3 ± 11.2 ^a^	161.7 ± 9.5 ^c^
Sherry lactone 1	89.3 ± 7.5 ^e^	235.7 ± 6.4 ^d^	458.7 ± 18.7 ^b^	394.0 ± 23.5 ^c^	1620.3 ± 15.0 ^a^
Sherry lactone 2	229.0 ± 14.9 ^d^	394.0 ± 8.7 ^c^	649.0 ± 28.1 ^b^	224.3 ± 11.2 ^d^	1931.7 ± 12.3 ^a^
Volatile phenols					
4-Ethylguaiacol	4.0 ± 2.6 ^b^	22.0 ± 1.0 ^a^	22.3 ± 3.8 ^a^	0.3 ± 0.6 ^b^	0.7 ± 0.6 ^b^
Vanillin	2.7 ± 0.6 ^b^	4.3 ± 1.2 ^b^	5.3 ± 0.6 ^b^	4.7 ± 1.5 ^b^	10.3 ± 1.5 ^a^
Miscellaneous					
*N*-(3-methylbutyl)acetamide	37.0 ± 2.0 ^c^	147.3 ± 8.5 ^b^	231.7 ± 8.3 ^a^	20.3 ± 3.1 ^c^	149.7 ± 9.3 ^b^

Different letters mean values are significantly different for *p* < 0.05 (ANOVA; Tukey’s HSD).

**Table 2:** Average score of 10 sensory descriptors of passito wines obtained from sound grapes (A-S and B-S) and noble-rotten grapes (A-N20, A-N40 and B-N50). Values (± standard deviation) are average of three independent trials.

	A-S	A-N20	A-N40	B-S	B-N50
Aroma intensity	6.6 ± 0.3 ^ab^	5.5 ± 0.6 ^c^	6.4 ± 0.4 ^b^	6.2 ± 0.3 ^b^	7.1 ± 0.4 ^a^
Floral	3.1 ± 0.3 ^b^	1.5 ± 0.5 ^d^	1.6 ± 0.2 ^d^	4.1 ± 0.2 ^a^	2.5 ± 0.3 ^c^
Almond	3.6 ± 0.5 ^a^	3.4 ± 0.3 ^a^	3.9 ± 0.3 ^a^	3.4 ± 0.2 ^a^	3.8 ± 0.5 ^a^
Honey	4.4 ± 0.3 ^d^	5.7 ± 0.2 ^b^	5.9 ± 0.4 ^b^	5.0 ± 0.1 ^c^	6.8 ± 0.1 ^a^
Figs	3.9 ± 0.2 ^b^	4.1 ±0.2 ^b^	5.4 ±0.3 ^a^	4.1 ± 0.2 ^b^	5.1 ± 0.3 ^a^
Apricot	2.9 ± 0.4 ^c^	3.4 ±0.3 ^c^	4.4 ± 0.3 ^b^	4.2 ± 0.1 ^b^	6.4 ± 0.4 ^a^
Vegetal	2.0 ± 0.3 ^b^	3.4 ±0.3 ^a^	3.1 ± 0.3 ^a^	1.2 ± 0.1 ^c^	1.5 ± 0.2 ^c^
Resinous	2.2 ± 0.2 ^b^	2.3 ±0.2 ^b^	2.8 ± 0.2 ^b^	2.5 ± 0.2 ^b^	4.7 ± 0.2 ^a^
Vanilla	2.7 ± 0.3 ^b^	3.1 ±0.3 ^ab^	2.6 ± 0.2 ^b^	3.5 ± 0.3 ^a^	3.6 ± 0.4 ^a^
Caramel	2.9 ± 0.3 ^b^	4.6 ±0.2 ^a^	4.5 ± 0.2 ^a^	2.9 ± 0.2 ^b^	4.9 ± 0.1 ^a^

Different letters mean values are significantly different for *p* < 0.05 (ANOVA; Tukey’s HSD).

## Supplementary Materials

The following are available online at https://www.mdpi.com/2304-8158/8/12/642/s1, Table S1: Content on ethanol, total dry extract, total acidity, and reducing sugars of five passito wines obtained from sound (A-S and B-S) and noble-rotten (A-N20, A-N40 and B-50) grapes, and the minimal parameters established by the regulation of production of Recioto di Soave DOCG and Recioto di Gambellara DOCG.
