# Supplementary material for: Correlating Noble Rot Infection of Garganega Withered Grapes with Key Molecules and Odorants of Botrytized Passito Wine
Source: Foods. 2019 Dec 4;8(12):642. doi: 10.3390/foods8120642 (PMC6963700; doi:10.3390/foods8120642)
Supplement: Supplementary file 1 [file foods-08-00642-s001.pdf]

Supplementary materials

Simonato et al. "Correlating noble rot infection of Garganega withered grapes with key molecules and odorants of botrytized passito wine".

Table S1. Content on ethanol, total dry extract, total acidity and reducing sugars of five passito wines obtained from sound (A-S and B-S) and noble-rotten (A-N20, A-N40 and B-50) grapes and the minimal parameters established by the regulation of production of Recioto di Soave DOCG and Recioto di Gambellara DOCG.

	**A-S**	**A-N20**	**A-N40**	**B-S**	**B-N50**	**Soave**	**Gambellara**
Ethanol (% v v^{-1})	13.1	13.4	13.8	14.0	14.7	12.0	11.3
Total dry extract (g L^{-1})	33	38	44	67	85	27	22
Total acidity (g L^{-1})	5.5	5.4	5.4	5.6	5.4	5.0	4.5
Reducing sugars (g L^{-1})	54	76	83	115	145	70	50
